# Quantitative assessment of myelination patterns in preterm neonates using T2-weighted MRI

**DOI:** 10.1038/s41598-019-49350-3

**Published:** 2019-09-10

**Authors:** Siying Wang, Christian Ledig, Joseph V. Hajnal, Serena J. Counsell, Julia A. Schnabel, Maria Deprez

**Affiliations:** 1 Institute of Biomedical Engineering, Department of Engineering Science, University of Oxford, Oxford, UK; 2 Department of Computing, Imperial College London, London, UK; 3 School of Imaging Sciences & Biomedical Engineering, King’s College London, London, UK

**Keywords:** Computational neuroscience, Biomedical engineering

## Abstract

Myelination is considered to be an important developmental process during human brain maturation and closely correlated with gestational age. Quantitative assessment of the myelination status requires dedicated imaging, but the conventional T_2_-weighted scans routinely acquired during clinical imaging of neonates carry signatures that are thought to be associated with myelination. In this work, we develop a quatitative marker of progressing myelination for assessment preterm neonatal brain maturation based on novel automatic segmentation method for myelin-like signals on T_2_-weighted magnetic resonance images. Firstly we define a segmentation protocol for myelin-like signals. We then develop an expectation-maximization framework to obtain the automatic segmentations of myelin-like signals with explicit class for partial volume voxels whose locations are configured in relation to the composing pure tissues via second-order Markov random fields. The proposed segmentation achieves high Dice overlaps of 0.83 with manual annotations. The automatic segmentations are then used to track volumes of myelinated tissues in the regions of the central brain structures and brainstem. Finally, we construct a spatio-temporal growth models for myelin-like signals, which allows us to predict gestational age at scan in preterm infants with root mean squared error 1.41 weeks.

## Introduction

Human brain maturation involves a complex series of morphological, structural and functional changes. Among these changes is the process of myelin growth and axon ensheathment known as myelination, which facilitates electrical conduction in the neural system^1^. Process of myelination is initiated during intra-uterine development, progressing from brainstem and deep structures, which are partly myelinated prior to birth, towards peripheral brain regions (e.g. cerebral white matter tracts) that mostly myelinate during the first year, and continue throughout life^1–4^. Patterns of myelination were initially observed through histological studies^2,5^ and more recently, T_1_- and T_2_-weighted Magnetic Resonance Imaging (MRI) has been established as the primary method for qualitative assessment of myelination during prenatal and early postnatal period^1,4^.

Preterm birth and other perinatal insults often lead to injury to immature WM and subsequent hypomyelination^6,7^. Impaired myelination in posterior limb of internal capsule (PLIC) is considered a sign of developmental delay in the newborn^8^ and is one of the cerebral abnormalities observed on MRI of preterm infants at term-equivalent age linked to very preterm birth^9^. Previous work has also suggested that absolute volume of myelinated white matter (WM) at term equivalent age is decreased in preterm infants^10^. Diffusion MRI has shown decrease in fractional anisotropy (FA) in PLIC in preterm infants at term equivalent age^11^ which may reflect either reduction in myelination or decreased coherence of WM fibers^12^. A recent study of a small cohort of preterm infants suggest that decrease in FA is accompanied by increase in T_2_ relaxation times in very preterm infants compared to late preterm infants at term equivalent age^13^. White matter abnormalities observed on MRI of preterm infants have been linked to neurodevelopmental impairment, such as motor and cognitive delay^14–16^.

Progressing myelination results in shortening of T_1_ and T_2_ relaxation times^1,3,17^. However, their time courses are different. T_1_ shortening starts already in “pre-myelination” stage and is thought to be linked to the bounding of free water molecules to the accumulating building blocks of myelin (choloesterol and glicolipids). On the other hand, shortening of T_2_ relaxation times seems to correspond to tightening of the myelin sheath around the axons and consequent redistribution of free water, reflecting true maturation of the myelin^3,17^. T_1_ and T_2_-weighted weighted MRI remain the most common modalities for imaging neonatal brain^18,19^ and qualitative descriptions of myelination using these modalities^20,21^ are consistent with histological observations^2^. Though specialised sequences for imaging of myelin have been proposed (e.g. myelin water imaging^22^, magnetization transfer imaging^23^), they are not routinely available and their application in preterm and neonatal population would currently be limited to small prospective studies. On the other hand, availability of quantitative markers of myelin using standard T_2_-weighted imaging would facilitate quantitative evaluation of mature myelin in large-scale studies of neurodeveoplment in preterm and neonatal populations.

In this study, we refer to the tissue that is likely to contain myelin in T_2_-weighted neonatal brain MRI as myelin-like signals (MLS). One way of developing a quantitative markers of myelination is to develop an automatic method for segmentation of MLS, that can be further utilised in volumetric studies and spatio-termporal modelling of progressing myelination. Automatic segmentation of MLS is challenging and there is currently a lack of dedicated methods for segmenting MLS during preterm and neonatal period. Recently developed methods for neonatal brain segmentation in the NeoBrainS12 challenge^24–28^ all showed great promise in segmenting a number of brain structures in neonatal brain MRI. However, none of them performed well in segmenting myelination^29^. Most neonatal brain segmentation methods adapt the approaches developed for adults^30–34^, and use a probabilistic atlas or manual annotations to obtain prior information on the expected tissue locations. However, myelin is not included in any of the existing neonatal brain atlases^35–37^ or manual annotation database^38^, and neither it is considered in segmentation protocol in Developing Human Connectomme Project^39^. Another issue that complicates accurate segmentation of MLS in perinatal period is its small volume compared to resolution of the MRI. Partial volume (PV) effect (two or more tissues mixing in a single voxel) therefore needs to be taken into account. Many methods for PV modeling were previously proposed^40–45^, however they all require prior information about location of mixing tissues, which is currently not available for MLS.

In this paper we propose an automatic method of MLS on T_2_-weighted neonatal brain images that does not require any probabilistic atlas or manual annotation of myelin. Similarly to Ledig *et al*.^41^ we model PV between MLS and backgournd (BKG) voxels using second-order Markov random fields (MRFs) within an expectation-maximization (EM) framework. We introduce an explicit PV class whose locations are configured in relation to MLS and BKG using a 3D connectivity tensor. We distinguish between MLS and other tissue with similar intensity ranges by defining an anatomical region of interest (ROI) for MLS based on anatomical knowledge. Our method achieves automatic MLS segmentations of high Dice coefficients (DCs)^46^ with respect to the manual annotations for 16 preterm infants at one-week intervals between 29 and 44 weeks gestational age (GA). The proposed segmentation method is applied to T_2_-weighted scans of 114 preterm infants to develop quantitative markers of myelination. Firstly, we perform volumetric analysis of the progressing myelination and show that myelination increases in deep brain region while it is stable in brainstem. Secondly, we build a spatio-temporal model of progressing myelination, which we compare to the qualitative studies of myelination^4,21^. Finally we show that the spatio-termporal atlas of progressing myelination enables us to predict gestational age at scan with high accuracy and is thus a potential quantitative marker of developmental delay.

## Results

### Volumetric analysis of progressing myelination

**Figure 1:**
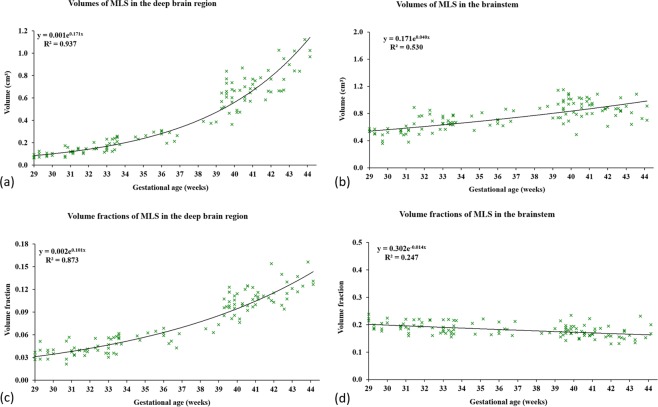
(**a**,**b**) Volumes of the automatic segmentations for myelin-like signals (MLS) in the deep brain region and brainstem obtained using the proposed segmentation method, (**c**,**d**) volume fractions of MLS plotted against the gestational ages at scan (GAs) of 114 subjects between 29 and 44 weeks GA. In deep brain region the MLS volume appears to grow exponentially with GA, and this trend persists in the MLS volume fraction after correcting for the different sizes of the deep brain region in individual subjects. In brainstem the trend for MLS volume appears nearly linear with GA, which results in an almost constant MLS volume fraction after correcting for the different sizes of the brainstem in individual subjects.

Automatic segmentation of the MLS was applied to 114 T_2_-weighted neonatal scans. The details of the subjects, acquisition protocol and the segmentation method can be found in the Methods section. The volumes calculated from the resulting segmentations in the deep brain region and brainstem were plotted against GA in Fig. 1. We fitted exponential trend lines with equation $y=a{e}^{bx}$ and calculated *R*^2^ scores.

We found that the MLS volume appeared to grow exponentially with GA in the deep brain region (${R}^{2}=0.94$), and this trend persisted in the MLS volume fraction after we corrected for the different deep brain region sizes in individual subjects (${R}^{2}=0.87$). In contrast, the MLS volume appeared to grow nearly linearly with GA in the brainstem (${R}^{2}=0.53$ for exponential model, ${R}^{2}=0.52$ for linear model), which resulted in an almost constant MLS volume fraction after correcting for the different brainstem sizes in individual subjects (${R}^{2}=0.25$ for exponential model, ${R}^{2}=0.24$ for linear; very small $b=-\,0.015$ suggest nearly constant or slightly negative trend). This is consistent with the previous findings that most of the brainstem is myelinated before 29 weeks GA, whereas new regions become myelinated in the deep brain between 29 and 44 weeks GA^21,47,48^. This suggest that the MLS volume increased in the brainstem mainly due to the overall brain growth, and the increase in the deep brain region was attributed to both brain growth and new myelination.

### Spatio-temporal model of progressing myelination

**Figure 2:**
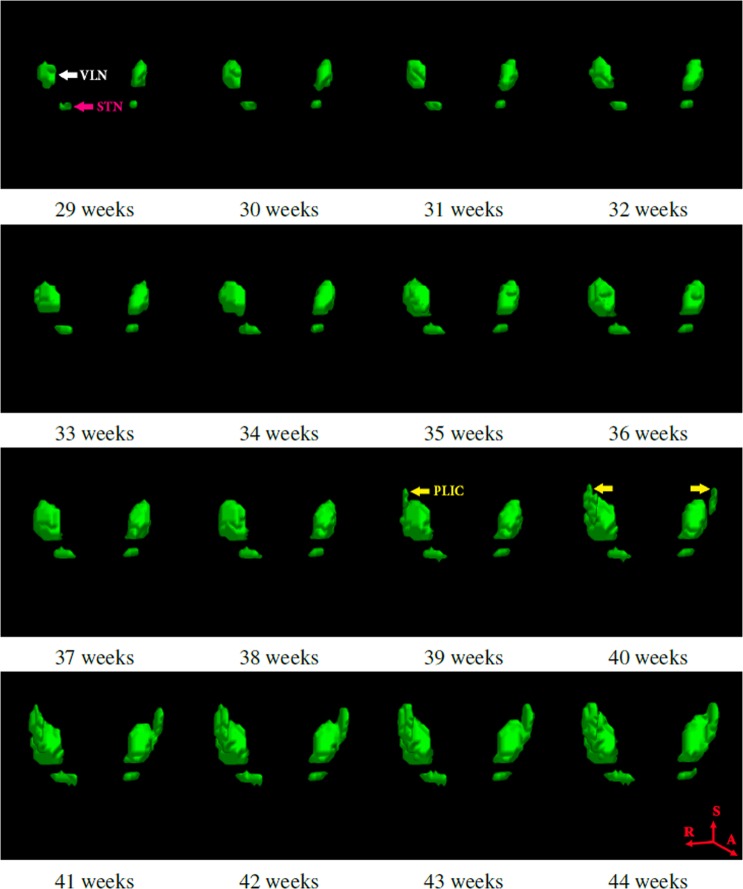
Spatio-temporal growth model for myelin-like signals (MLS) in the deep brain region between 29 and 44 weeks GA. The ventrolateral nuclei (VLN) and subthalamic nuclei (STN) appear to be myelinated before 29 weeks GA. MLS becomes evident in both tracts of the posterior limbs of the internal capsule (PLIC) at approximately 40 weeks GA. This is an important landmark for evaluating neonatal brain development. As the different deep brain region sizes in individual subjects have been corrected through non-rigid registration, the progression of MLS is completely due to the appearance of new myelination without contribution from the overall brain growth.

Segmented images were registered into common reference space, and we fitted a modified logistic function to each voxel location to create spatio-temporal model of progressing myelination. The resulting model for MLS in deep brain region is presented in Fig. 2. The details of the spatio-temporal modelling can be found in Methods section and our previous work^49^. Note that because we corrected for the different sizes of each ROI in individual subjects through non-rigid registration^50^, the MLS progression depicted by the spatio-temporal model was entirely due to the appearance of new myelinated brain structures without contribution from the overall brain growth.

We found that there was an evident difference in the onset and rate of myelination in deep brain region and brainstem. The model of MLS in deep brain region (Fig. 2) showed that the ventro-lateral nuclei (VLN) and sub-thalamic nucleus (STN) were myelinated before 29 weeks GA. Moreover, the model captured the arrival of MLS in both of the posterior limbs of internal capsule (PLIC) at 40 weeks GA, which confirmed the clinically observed time point^4,21^. Myelination of the PLIC tracts is an important landmark for evaluating neonatal brain development^51^. The brainstem, however, appeared to be well developed by the time of 29 weeks GA, and there was no new myelination in the age range of our investigation. We summarise these milestones in Table 1, and compare them to the previous studies by Counsell *et al*.^21^ and Barkovich *et al*.^4^.

**Table 1:** Comparison of myelination milestones between the spatio-temporal atlas (Myelin atlas) and the qualitative description^4,21^.

Region	structures	Counsell *et al*.	Barkovich *et al*.	Myelin atlas
Brainstem	SCP, DSCP, IC, LL, ML, MLF, VN	25–30 weeks GA	25–30 weeks GA	≤29 weeks GA
Deep brain	VLN	25 weeks GA	—	≤29 weeks GA
Deep brain	STN	28 weeks GA	—	≤29 weeks GA
Deep brain	PLIC	40 weeks GA	40 weeks GA	39–40 weeks GA

The milestone refers to first time-point in gestation when the myelin is identified in a new site. The full names of the structures are provided in the section “Segmentation Protocol”.

### Estimation of gestational ages

The spatio-temporal models of MLS were utilised for estimation of gestational ages at scan (GA), to evaluate whether MLS were suitable markers of GA. The individual segmentations registered to the reference space were compared with models for each time-point using sum of squared errors and the GA with smallest error between the model and the segmentation was chosen as the final GA estimate for each subject.

**Figure 3:**
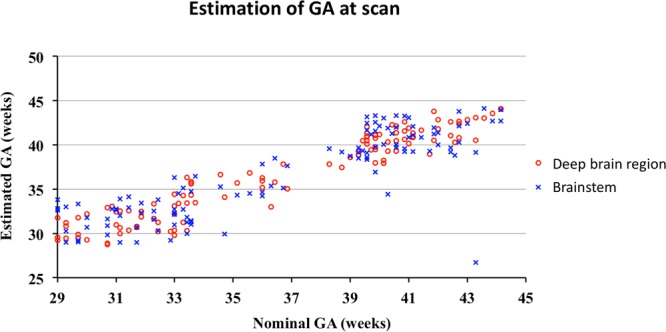
Gestational ages (GAs) predicted for 114 preterm infants and compared to the nominal GAs using the logistic growth models for myelin-like signals (MLS) in the deep brain region and brainstem. The predictions based on MLS in the brainstem display larger deviations from the nominal GAs than in the deep brain region. This is because most of the MLS spatio-temporal changes between 29 and 44 weeks GA occur in the deep brain region, whereas the brainstem provides much less information on progressing myelination during this particular age period.

The estimated GAs were plotted against the nominal values in Fig. 3. We obtained root mean squared errors (RMSEs) of 1.41 weeks and 2.56 weeks in the deep brain region and brainstem respectively. Therefore, each ROI has a different predictive power that best assesses a particular period of brain development. The the deep brain region with the more prominent MLS growth between 29 and 44 weeks GA produced the more accurate age estimates for preterm infants in this age range.

## Discussion

In this work we proposed quantitative markers of the myelination patters in preterm neonates with GA between 29 and 44 weeks using T_2_-weighted MRI. We have proposed a novel dedicated method for segmentation of myelin-like signals with high reproducibility comparable to the intra-rater variability. The achieved Dice overlap with manual segmentations 0.83 can be considered particularly high given small size of myelinated structures compared to the voxels size. This was achieved by defining a clear segmentation protocol and designing a dedicated method to reproduce the protocol reliably. We have used Gaussian Mixture Model (GMM) to estimate the intensity profiles of myelinated and non-myelinated tissues to overcome the problem of qualitative nature T_2_-weighted MRI. Modelling of partial volume effect proved to be particularly important, as GMM alone did not produce reliable results. On the other hand, modeling the partial volume by an extra class and constraining its location using Markov Random Fields resulted in a stable segmentation method. We have demonstrated, that resulting segmentations can be used to produce quantitative markers of myelination by calculating volumes of myelinated tissues in absolume terms (e.g. in mL) or as a proportion of a pre-defined region of interest, to account for individual differences in brain sizes. Additionally, modelling of typical patterns of myelination during this age-range and accurate method for estimation of myelination status in form of predicted GA has been proposed. This method might have potential in uncovering developmental delays in this age group. Other studies have explored myelination paterns in infancy and childhood^52–56^, but this is the first such detailed study during preterm neonatal period.

Inherent limitation of any study of myelination patters stems from lack of specificity of MRI sequences towards myelin. Histological studies consistently demonstrate that many MRI sequences (including quantitative MRI such as relaxometry and MTI) are sensitive to myelin, but none is specific, which means that intensity patterns linked to degree of myelination could also be attributed to other underlying biological processes^1^. We have overcome this issue by defining a segmentation protocol based on the available literature^21^ and creating a ROI “deep brain region” to separate myelinating structures from the structures that are more likely to be composed of densely packed grey matter. Inherently, uncertainties in the existing anatomical knowledge also exist in the proposed segmentation methodology. Nevertheless, gradual increase of the MLS in our spatio-termporal model supports the hypothesis that we are indeed detecting developing myelin.

Interesting direction for future work would be to quantify myelination progress using combination of T_1_ and T_2_-weighted imaging. Given that shortening of T1 and T2 relaxation times determine different stages of myelination process^17^, their joint analysis could provide more detailed staging of myelination of different brain structures^56^, resulting in further insights into brain maturation during perinatal period.

Though our segmentation method offers quatitative measure of volume of myelinated tissue, it cannot determine proportion of myelin within each voxel. Quantitative methods such as relaxometry and MTI could produce even more accurate quantification of myelin maturation once large databases in this age-range become available. Our results however show that the proposed methods can be nevertheless a useful marker of brain maturation during this period of brain development and has particular applications in large-scale studies to determine consequences of preterm birth on developing myelin.

## Methods

### Subjects and MR acquisition

114 T_2_-weighed scans of 95 preterm infants were acquired between 29 and 44 weeks GA at Hammersmith Hospital, London, UK. Ethical permission for this study was granted by the Hammersmith and Queen Charlotte’s and Chelsea Research Ethics Committee (07/H0707/101). Informed written parental consent was obtained prior to imaging. All methods and experiments were performed in accordance with relevant guidelines and regulations. T_2_-weighted fast spin-echo brain images were acquired on a 3 T Philips Intera system with repetition time = 8700 ms, echo time = 160 ms and voxel sizes = 0.86 mm × 0.86 mm × 1 mm. Full information about the subjects is given in Table 2.

**Table 2:** Subjects and scans in the study.

	Subjects	All scans	Preterm scans	Term scans
Number	95	114	58	56
GA scan (weeks)		36.6 ± 4.6	32.4 ± 2.2	40.9 ± 1.5
GA birth (weeks)	29.6 ± 2.6	29.5 ± 2.6	29.4 ± 2.3	29.7 ± 2.9
Birth weight (kg)	1.27 ± 0.52	1.26 ± 0.48	1.20 ± 0.33	1.33 ± 0.61
Female	48	58	32	26
Male	47	56	26	30
Singletons	37	50	27	23
Twins	58	64	31	33
WM lesions	1	2	1	1
WM cysts	2	2	1	1
Cerebellar heam orrage	1	1	1	0

Some subjects had two or three scans. The first column refers to numbers and averages over all subjects. The other columns refer to numbers or averages over all scans, scans at preterm period (GA at scan between 27 and 37 weeks) and term equivalent age (GA at scan between 37 and 44 weeks).

### Segmentation protocol

**Figure 4:**
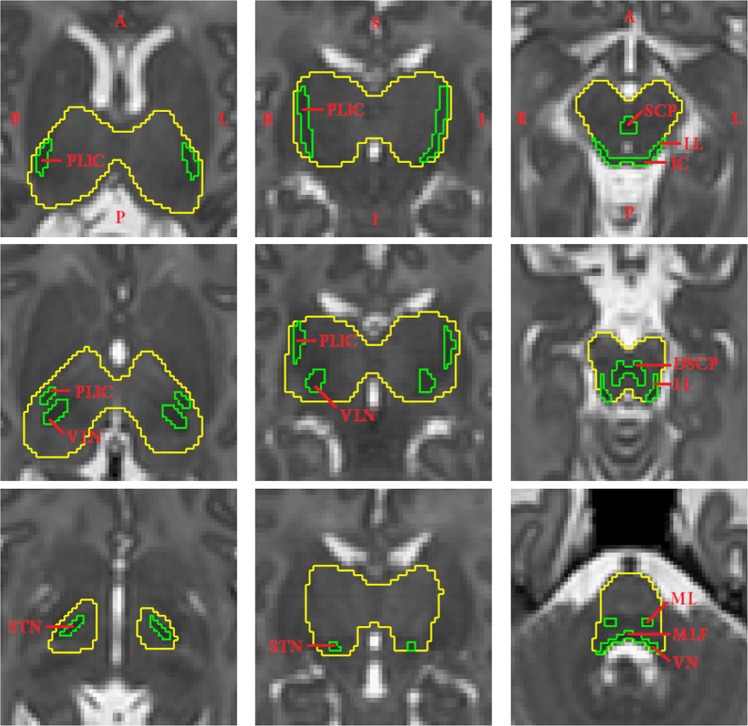
Manual annotations of myelin-like signals (green label) in the deep brain region and brainstem of a subject at 42 gestational weeks, delineated according to the defined segmentation protocol. The regions of interest are labeled in yellow. The columns from left to right show the deep brain region in the axial and coronal views, and the brainstem in the axial view. Abbreviations: PLIC–posterior limb of the internal capsule, VLN–ventrolateral nucleus, STN–subthalamic nucleus, SCP–superior cerebellar peduncle, DSCP–decussation of the superior cerebellar peduncle, IC–inferior colliculus, LL–lateral lemniscus, ML–medial lemniscus, MLF–medial longitudinal fasciculus, VN–vestibular nucleus.

Our segmentation protocol focuses on central brain structures (thalamus, basal ganglia and internal capsule) and brainstem for assessing MLS because in these regions the myelination is present between 29 and 44 weeks GA^21,48^. Cerebellum was not included in this study. Since the myelinated nuclei can have similar intensities to the densely organized GM in the basal ganglia which are thought not to contain myelin between 29 and 44 weeks GA^21^, we create a custom ROI containing the posterior limbs of the internal capsule (PLIC), ventrolateral nuclei (VLN) and subthalamic nuclei (STN), referred to as deep brain region in this paper. The MLS segmentation protocol is defined based on both image intensities and anatomical knowledge^21^. We delineate three structures, PLIC, VLN and STN in the the deep brain region. In the brainstem we define the segmentation protocol mainly based on intensities because the image resolution is insufficient to identify the detailed anatomical structures. An example of the segmentation protocol is shown in Fig. 4.

### Segmentation pipeline

**Figure 5:**
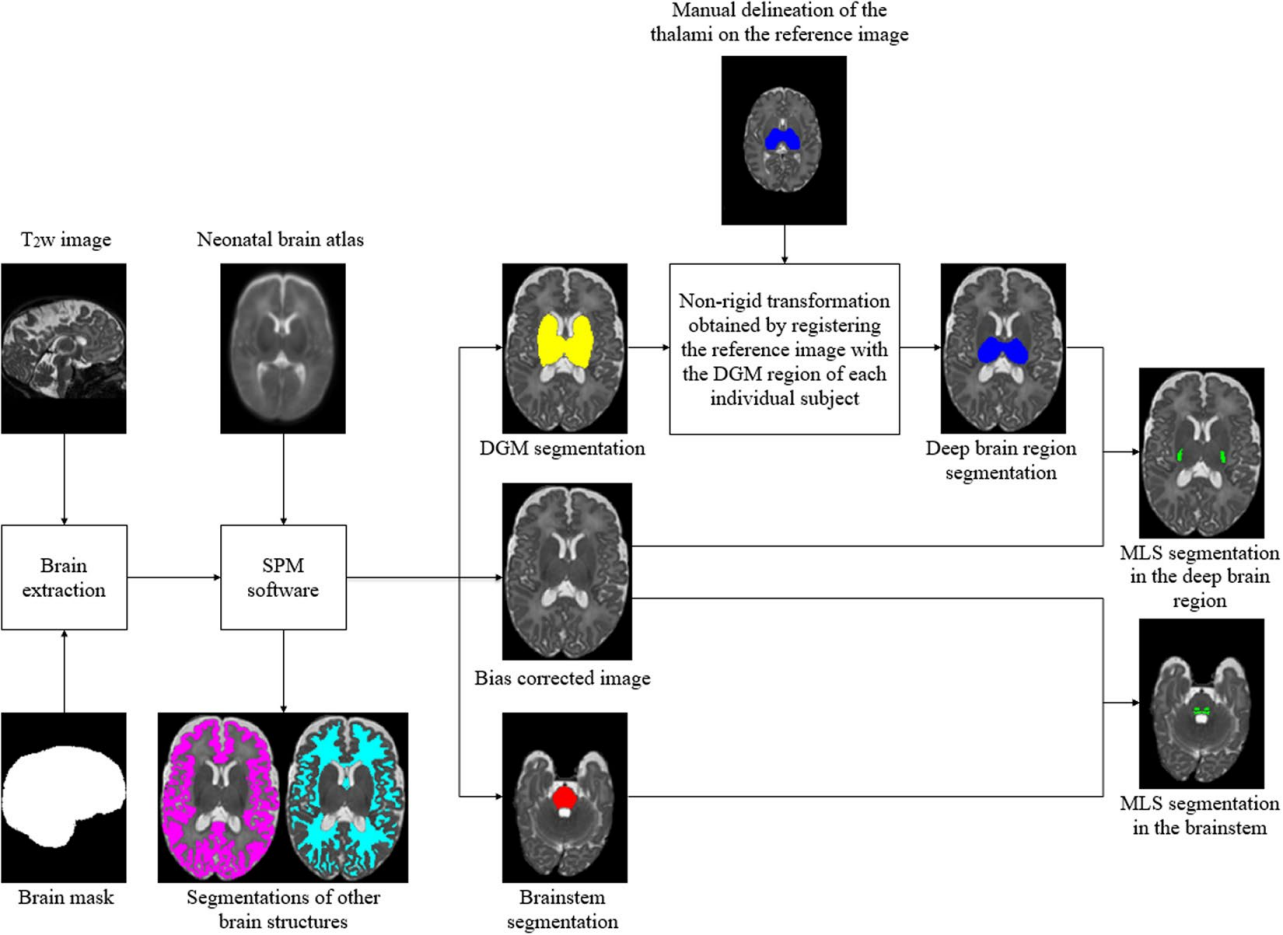
Schematic flow of the proposed segmentation approach for myelin-like signals (MLS) on T_2_-weighted neonatal brain images. Through image preprocessing, we achieve brain extraction and bias field correction, and obtain the binary masks of the deep brain region and brainstem for individual subjects. The automatic segmentations of the deep gray matter (DGM), obtained using the Statistical Parametric Mapping (SPM) software, contain the basal ganglia, thalami, hippocampi and amygdalae. We extract the masks for the deep brain region from the DGM segmentations via label propagation.

The overall pipeline proposed for MLS segmentation on T_2_-weighted neonatal brain MR images is shown in Fig. 5. The images are first pre-processed to achieve brain extraction and bias field correction, and to obtain the binary ROI masks of the deep brain region and brainstem for individual subjects. We remove the non-brain tissues using label propagation^31^ of manually annotated brain masks, and segment the skull-stripped images using the Statistical Parametric Mapping (SPM) software (www.fil.ion.ucl.ac.uk/spm, version SPM8)^30^. The spatial priors are provided by a publicly available (brain-development.org/brain-atlases) 4D probabilistic neonatal brain atlas^35^. Subsequently, we obtain the bias-corrected images as well as the segmentations of the cortical GM, WM, deep gray matter (DGM), brainstem, cerebellum and CSF.

We use the automatic SPM segmentations of the brainstem directly as the ROI mask. The DGM segmentations include the basal ganglia, thalami, hippocampi and amygdalae. Since the myelinated nuclei can have similar intensities to the densely organized GM in the basal ganglia which is considered not to contain myelin between 29 and 44 weeks GA^21^, we extract the ROI masks for the deep brain region from the DGM segmentations in order to prevent misclassifications by the intensity-based segmentation model. We first manually delineate the deep brain region on the T_2_-weighted image of a single reference subject at 36 weeks GA. We register the reference image with the dilated DGM region of each subject using free-form deformation (FFD) non-rigid registration^50^, and then transform the manual delineation of the deep brain region from the reference space to each individual subject’s space.

### Segmentation of myelin like signals

The segmentation of myelin like signals is performed in Expectation Maximisation framework. We model three classes, MLS, BKG and PV, with their intensities defined by a Gaussian distribution $G(y,{\mu }_{k},{\sigma }_{k})$ with means *μ*_*k*_ and variances ${\sigma }_{k}^{2}$, where *y* is the voxel intensity and *k* is the class index. We approximate the PV class mean as the arithmetic mean of the composing pure tissue means *μ*_*MLS*_ and *μ*_*BKG*_, and assume that the variances of all three classes are identical:

1 $${\mu }_{PV}=\frac{1}{2}({\mu }_{MLS}+{\mu }_{BKG})$$

2 $${\sigma }_{MLS}^{2}={\sigma }_{PV}^{2}={\sigma }_{BKG}^{2}$$

By substituting Eqs. 1 and 2 into the objective function of log likelihood^57^, the ML estimations for the means of the MLS and BKG classes result in the following system of linear equations (see more details in the appendix):

3 $$[\begin{array}{c}{\mu }_{{\rm{MLS}}}^{(m+1)}\\ {\mu }_{{\rm{BKG}}}^{(m+1)}\end{array}]={[\begin{array}{cc}\frac{1}{4}{B}_{{\rm{PV}}} & {B}_{{\rm{BKG}}}+\frac{1}{4}{B}_{{\rm{PV}}}\\ {B}_{{\rm{MLS}}}+\frac{1}{4}{B}_{{\rm{PV}}} & \frac{1}{4}{B}_{{\rm{PV}}}\end{array}]}^{-1}\,[\begin{array}{c}{A}_{{\rm{BKG}}}+\frac{1}{2}{A}_{{\rm{PV}}}\\ {A}_{{\rm{MLS}}}+\frac{1}{2}{A}_{{\rm{PV}}}\end{array}]$$

where

$${A}_{k}=\mathop{\sum }\limits_{i=1}^{N}\,{y}_{i}{p}_{ik}^{(m+\mathrm{1)}}\,{\rm{and}}\,{B}_{k}=\mathop{\sum }\limits_{i=1}^{N}\,{p}_{ik}^{(m+\mathrm{1)}}$$

The variances ${\sigma }_{k}^{2}$ of class *k* are updated as

4 $${({\sigma }_{k}^{(m+1)})}^{2}={({\sigma }^{(m+1)})}^{2}=\frac{1}{N}\,\mathop{\sum }\limits_{l=1}^{K}\,\mathop{\sum }\limits_{i=1}^{N}\,{p}_{il}^{(m+1)}{({y}_{i}-{\mu }_{l}^{(m+\mathrm{1)}})}^{2}$$

where *p*_*ik*_ is the posterior probability of voxel *i* belonging to class *k*, *y*_*i*_ is the observed intensity of voxel *i*, *K* the number of classes, and *m* the iteration number. The above equations comprise the M-step of Expectation Maximisation algorithm.

The E-step of the expectation maximisation algorithm calculates the posterior probabilies, which represent probabilistic segmentation. The probabilistic segmentation is regularised with the spatial prior $p({{\boldsymbol{z}}}_{{\boldsymbol{i}}}={{\boldsymbol{e}}}_{{\boldsymbol{k}}}|{{\boldsymbol{p}}}_{{{\mathscr{N}}}_{i}}^{(m)},{\Phi }_{z})$ determined from the posteriors of the neighboring voxels as described in^57^:

5 $${p}_{ik}^{(m+1)}=\frac{G({y}_{i},{\mu }_{k}^{(m)},{\sigma }_{k}^{(m)})p({{\boldsymbol{z}}}_{{\boldsymbol{i}}}={{\boldsymbol{e}}}_{{\boldsymbol{k}}}|{{\boldsymbol{p}}}_{{{\mathscr{N}}}_{i}}^{(m)},{\Phi }_{z})}{{\sum }_{k^{\prime} =1}^{K}\,G({y}_{i},{\mu }_{k^{\prime} }^{(m)},{\sigma }_{k^{\prime} }^{(m)})p({{\boldsymbol{z}}}_{{\boldsymbol{i}}}={{\boldsymbol{e}}}_{{\boldsymbol{k}}^{\prime} }|{{\boldsymbol{p}}}_{{{\mathscr{N}}}_{i}}^{(m)},{\Phi }_{z})}$$

where

6 $$p({{\boldsymbol{z}}}_{{\boldsymbol{i}}}={{\boldsymbol{e}}}_{{\boldsymbol{k}}}|{{\boldsymbol{p}}}_{{{\mathscr{N}}}_{i}}^{(m)},{\Phi }_{z})=\frac{{e}^{-{U}_{{\rm{MRF}}}({{\boldsymbol{e}}}_{{\boldsymbol{k}}}|{{\boldsymbol{p}}}_{{{\mathscr{N}}}_{i}}^{(m)},{\Phi }_{z})}}{{\sum }_{k^{\prime\prime} =1}^{K}\,{e}^{-{U}_{{\rm{MRF}}}({{\boldsymbol{e}}}_{{\boldsymbol{k}}^{\prime\prime} }|{{\boldsymbol{p}}}_{{{\mathscr{N}}}_{i}}^{(m)},{\Phi }_{z})}}$$

The symbol ***z***_***i***_ indicates to which class voxel *i* belongs, and ***e***_***k***_ is a unit vector of length *K* whose components are equal to zero except the *k*th component. The probabilities in the 26-neighborhood ${{\mathscr{N}}}_{i}$ of voxel *i* are denoted as ${{\boldsymbol{p}}}_{{{\mathscr{N}}}_{i}}$, and the MRF parameters as ${\Phi }_{z}$. We compute the energy function ${U}_{{\rm{MRF}}}({{\boldsymbol{e}}}_{{\boldsymbol{k}}}|{{\boldsymbol{p}}}_{{{\mathscr{N}}}_{i}},{\Phi }_{z})$ for second-order MRFs as follows:

7 $$\begin{array}{rcl}{U}_{{\rm{MRF}}}({{\boldsymbol{e}}}_{{\boldsymbol{k}}}|{{\boldsymbol{p}}}_{{{\mathscr{N}}}_{i}}^{(m)},{\Phi }_{z}) & = & \mathop{\sum }\limits_{{k}_{1}=1}^{K}\,{v}_{{k}_{1}}^{(m)}\,\mathop{\sum }\limits_{{k}_{2}\mathrm{=1}}^{K}\,{{\bf{T}}}_{k}({k}_{1},{k}_{2}){v}_{{k}_{2}}^{(m)}\\  & = & [{v}_{1}\,{v}_{2}\,\cdots \,{v}_{K}]\,[\begin{array}{cccc}{T}_{k}(1,1) & {T}_{k}(1,2) & \cdots  & {T}_{k}(1,K)\\ {T}_{k}(2,1) & {T}_{k}(2,2) & \cdots  & {T}_{k}(2,K)\\ \vdots  & \vdots  & \ddots  & \vdots \\ {T}_{k}(K,1) & {T}_{k}(K,2) & \cdots  & {T}_{k}(K,K)\end{array}]\,[\begin{array}{c}{v}_{1}\\ {v}_{2}\\ \vdots \\ {v}_{K}\end{array}]\end{array}$$

where

8 $${v}_{k}^{(m)}=\sum _{j\in {N}_{i}}\,\frac{{p}_{jk}^{(m)}}{{d}_{ij}}$$

Here *p*_*jk*_ is the probability that neighbor *j* belongs to class *k*, and *d*_*ij*_ the Euclidean distance between voxel *i* and neighbor *j*. The element of the connectivity tensor, denoted as ${{\bf{T}}}_{k}({k}_{1},{k}_{2})$, indicates the penalty when classes *k*_1_ and *k*_2_ are both present in the neighborhood of class *k*. By assigning a large value to ${{\bf{T}}}_{k}({k}_{1},{k}_{2})$ in Eq. 7, we are able to penalize a particular configuration when the probabilities of classes *k*_1_ and *k*_2_ are both high in the neighborhood. This would result in a large value of the energy function ${U}_{{\rm{MRF}}}({{\boldsymbol{e}}}_{{\boldsymbol{k}}}|{{\boldsymbol{p}}}_{{{\mathscr{N}}}_{i}},{\Phi }_{z})$, making voxel *i* less probable to belong to class *k*.

We assign the penalties ${{\bf{T}}}_{k}({k}_{1},{k}_{2})$ according to the following rules based on the triplet that contains class *k* of the current voxel and classes *k*_1_ and *k*_2_ of a pair of neighboring voxels:

- ${{\bf{T}}}_{k}({\rm{MLS}},{\rm{MLS}})$ and ${{\bf{T}}}_{k}({\rm{BKG}},{\rm{BKG}})$: We set the penalty to 0 if class *k* is the same as the pair. We assign a penalty *t*_1_ if *k* is a pure tissue class different from the pair to encourage the PV class as MLS and BKG are both in the triplet. We assign a penalty *t*_3_ if *k* is PV as there is no evidence for PV in the triplet. This effectively prevents over-estimation of the PV class.
- ${{\bf{T}}}_{k}({\rm{MLS}},{\rm{BKG}})$: We assign a penalty *t*_2_ if *k* is MLS or BKG, and set the penalty to 0 if *k* is PV. We aim to encourage the PV class by deliberately penalizing the MLS and BKG classes if they are both present in the neighborhood.
- ${{\bf{T}}}_{k}({\rm{MLS}},{\rm{PV}})$, ${{\bf{T}}}_{k}({\rm{PV}},{\rm{PV}})$ and ${{\bf{T}}}_{k}({\rm{BKG}},{\rm{PV}})$: We set the penalty to 0 regardless of the class of *k*.

The values of penalties *t*_1_, *t*_2_ and *t*_3_ needed to be determined empirically and were set to 0.05, 0.03 and 0.01 by optimising the agreement with manual segmentation using Dice score^46^.

The EM algorithm is initialized by assigning 6 percent voxels with lowest intensities to MLS and rest to BKG class in deep brain region, while 25th percentile is used for brainstem. These values were estimated from manual segmentations. Tissue classification is achieved by interleaving the E-step and M-step until the relative change in the objective function of log likelihood^57^ is less than 0.01%. The resultant PPMs are converted into hard segmentations using the maximum-vote rule. In addition, we reclassify the PV voxels as one of the composing pure tissues by calculating the fraction of the MLS class, denoted as *f*_*i*_, at PV voxel *i*:

9 $${f}_{i}=\frac{{\mu }_{{\rm{BKG}}}-{y}_{i}}{{\mu }_{{\rm{BKG}}}-{\mu }_{{\rm{MLS}}}}$$

where *μ*_MLS_ and *μ*_BKG_ are the means of the MLS and BKG classes, and *y*_*i*_ the observed intensity. PV voxels with fractions above 0.5 are reclassified as MLS and combined with the voted hard segmentation of the MLS class to form the final segmentation.

### Evaluation of automatic segmentation

The automatic segmentation method of MLS was evaluated against manual annotations of MLS for 16 preterm infants, each at a different time point, between 29 and 44 weeks GA at one-week intervals. Eight of the 16 subjects, from 30 weeks GA onward at two-week intervals, have a repeated manual segmentation delineated by the same rater in order to assess the intra-rater reliability. The quality of these manual annotations was confirmed by a clinical expert.

We compared agreement with manual segmetations for three different methods: Gaussian Mixture Model with 2 classes MLS and BKG (GMM), Gaussian Mixture Model with explicit PV modelling but no spatial regularisation (GMM-PV) and the proposed three class model with spatial regularisation using MRF (GMM-PV-MRF). The proposed method clearly outperformed the other methods as well as the initial tresholding as shown in Tables 3 and 4. Additionally, the proposed method GMM-PV-MRF was found to be insensitive to the initialisation, while other tested method depended on correct initial threshold.

**Table 3:** Average Dice coefficients (DCs) (±standard deviations) for automatic segmentations of myelin-like signals in deep brain region compared with manual segmentations for 16 test subjects aged between 29 and 44 gestational weeks.

Method	Average DC	*p*-value
Thresholding at the 6th percentile	0.789 ± 0.091	0.043
GMM	0.762 ± 0.092	0.001
GMM-PV	0.662 ± 0.192	0.002
GMM-PV-MRF	0.837 ± 0.057	—

GMM-PV-MRF is compared with all the other methods using two-tailed Student’s *t*-tests at the 5% significance level.

**Table 4:** Average Dice coefficients (DCs) (±standard deviations) for automatic segmentations of myelin-like signals in brainstem compared with manual segmentations for 16 test subjects aged between 29 and 44 gestational weeks.

Method	Average DC	*p*-value
Thresholding at the 25th percentile	0.827 ± 0.035	0.020
GMM	0.675 ± 0.206	0.010
GMM-PV	0.762 ± 0.057	5.449 × 10^−4^
GMM-PV-MRF	0.831 ± 0.038	—

GMM-PV-MRF is compared with all the other methods using two-tailed Student’s *t*-tests at the 5% significance level.

We also assessed the intra-rater reliability as DCs between two sets of manual annotations that were available for eight of the 16 test subjects at two-week intervals between 30 and 44 weeks GA. We evaluated the automated model-based methods with respect to each manual set. The average DCs over the eight subjects are shown in Tables 5 and 6 along with the *p*-values of two-tailed Student’s *t*-tests with the intra-rater DCs at the 5% significance level. It can be seen that only GMM-PV-MRF performed equally well as a human rater in both ROIs, and demonstrated no significant differences with respect to the repeated manual annotations.

**Table 5:** Average Dice coefficients (DCs) (±standard deviations) for automatic segmentation of myelin-like signals in deep brain region of eight test subjects with respect to two sets of manual annotations compared to the intra-rater reliability.

Method	Manual set 1		Manual set 2		Intra-raterreliability
	Average DC	*p*-value	Average DC	*p*-value	
GMM	0.785 ± 0.085	0.257	0.754 ± 0.054	0.023	0.834 ± 0.043
GMM-PV	0.662 ± 0.210	0.060	0.626 ± 0.217	0.028	
GMM-PV-MRF	0.843 ± 0.051	0.624	0.816 ± 0.046	0.413	

The *p*-values indicate the outcomes of two-tailed Student’s *t*-tests with the intra-rater DCs at the 5% significance level. The performance of GMM-PV-MRF is close to the intra-rater reliability.

**Table 6:** Average Dice coefficients (DCs) (±standard deviations) for automatic segmentation of myelin-like signals in brainstem of eight test subjects with respect to two sets of manual annotations compared to the intra-rater reliability.

Method	Manual set 1		Manual set 2		Intra-rater reliability
	Average DC	*p*-value	Average DC	*p*-value	
GMM	0.702 ± 0.171	0.046	0.695 ± 0.156	0.026	0.842 ± 0.034
GMM-PV	0.763 ± 0.047	0.002	0.766 ± 0.077	0.031	
GMM-PV-MRF	0.832 ± 0.034	0.608	0.815 ± 0.024	0.087	

The *p*-values indicate the outcomes of two-tailed Student’s *t*-tests with the intra-rater DCs at the 5% significance level. The performance of GMM-PV-MRF is close to the intra-rater reliability.

We achieved high Dice overlaps of 0.83 comparable to the intra-rater variability. This proves that our method is reliable and reproducible across the target age-range. In contrast, the Dice overlap achieved by the methods that entered Neonatal Segmentation Challenge^29^, achieved Dice overlap 0.16–0.69 for 30 weeks GA and 0.23–0.68 for 40 weeks GA. None of the method achieved Dice overlap over 0.6 for both age groups.

### Spatio-temporal growth models for MLS

We constructed MLS growth models in the deep brain region and brainstem using voxelwise logistic regression based on the automatic segmentations computed using GMM-PV-MRF for the 114 subjects^49^. First we registered the T_2_-weighted image of each individual subject with the dilated ROI of the single reference subject at 36 weeks GA using FFD non-rigid registration^50^. This was the same reference subject used to create the ROI masks of the deep brain region. We used normalized mutual information (NMI) as the similarity measure and 10 mm B-spline control point spacing. The automatic MLS segmentations were then transformed accordingly from each subject’s space to the common reference space. Lastly, we constructed the growth model in each ROI by fitting a voxelwise logistic function to the transformed segmentations. Details of constructing of the spatio-temporal models can be found in our previous work^49^.

We further used the MLS growth models in the deep brain region and brainstem to predict GAs of the 114 preterm infants. The age estimates were determined by minimizing the sum of squared differences (SSD) between each individual transformed segmentation and the average growth model constructed in a leave-one-out procedure.
